# Becoming a general practitioner - Which factors have most impact on career choice of medical students?

**DOI:** 10.1186/1471-2296-12-25

**Published:** 2011-05-09

**Authors:** Kathrin Kiolbassa, Antje Miksch, Katja Hermann, Andreas Loh, Joachim Szecsenyi, Stefanie Joos, Katja Goetz

**Affiliations:** 1 Department of General Practice and Health Services Research, University Hospital Heidelberg, Germany; 2 Competence Centre General Practice, Baden-Wuerttemberg, Germany; 3 Department of General Practice, University Hospital Freiburg, Germany

## Abstract

**Background:**

In Germany, there is a shortage of young physicians in several specialties, the situation of general practitioners (GP) being especially precarious. The factors influencing the career choice of German medical students are poorly understood. This study aims to identify factors influencing medical students' specialty choice laying a special focus on general practice.

**Methods:**

The study was designed as a cross-sectional survey. In 2010, students at the five medical schools in the federal state of Baden-Wuerttemberg (Germany) filled out an online-questionnaire. On 27 items with 5-point Likert scales, the students rated the importance of specified individual and occupational aspects. Furthermore, students were asked to assign their intended medical specialty.

**Results:**

1,299 students participated in the survey. Thereof, 1,114 students stated a current choice for a specialty, with 708 students choosing a career in one of the following 6 specialties: internal medicine, surgery, gynaecology and obstetrics, paediatrics, anaesthetics and general practice. Overall, individual aspects ('Personal ambition', 'Future perspective', 'Work-life balance') were rated as more important than occupational aspects (i.e. 'Variety in job', 'Job-related ambition') for career choice. For students favouring a career as a GP individual aspects and the factor 'Patient orientation' among the occupational aspects were significantly more important and 'Job-related ambition' less important compared to students with other specialty choices.

**Conclusions:**

This study confirms that future GPs differ from students intending to choose other specialties particularly in terms of patient-orientation and individual aspects such as personal ambition, future perspective and work-life balance. Improving job-conditions in terms of family compatibility and work-life balance could help to increase the attractiveness of general practice. Due to the shortage of GPs those factors should be made explicit at an early stage at medical school to increase the number of aspirants for general practice.

## Background

In times of physicians' shortage it is important to know the interests and expectations of future physicians' generation which are essential for their career choice. In Germany, different medical disciplines are affected by shortage to various degrees [1]. The proportion of specialists increased from 45% to 52% from 1996 to 2008, whereas at the same time the proportion of general practitioners (GPs) decreased from 55% to 48% [2]. This led to 2,030 medical offices for GPs being vacant all over the country at the beginning of 2009 [2]. Age distribution of GPs aggravates the problem: 7,167 GPs were working in the federal state of Baden-Wuerttemberg (BW) in 2009 of which 23% were over 60 years of age [3]. The number of those who completed residency has decreased from 4,828 in 1995 to 1,168 in 2009 [4]. Most of the GPs in Germany are self-employed (94%) and only few are salaried (6%) [4].

Especially in rural areas those developments have led to a shortage of GPs and GPs who want to retire have problems to find a successor for their practices [3]. Young physicians cannot imagine professional life in a rural area for different reasons [5-7] i.e. the greater challenge to manage family responsibilities [7] including child care as well as finding an adequate school or the possibilities of leisure activities. Since the majority of young physicians want to start a family [8], family responsibilities have impact on career choice [7]. Those young physicians interested in working in general practice are more often female [9-14], of older age [15,16], less ambitious concerning career possibilities [17], they prefer a close physician-patient-relationship [9,10,18-21] and income and prestige are not their first priority [17]. While in many other countries a lot of research has been done to explore career choice, in Germany, research on factors influencing career choice is just at the beginning. A currently published study showed that physicians, who are female, grew up in rural areas, live with a partner and have children more likely aspire to become a GP [22]. However, there have been no comparisons so far with students interested in different medical specialties. Moreover, to attract more young doctors to the field of general practice it is essential, to identify relevant aspects influencing students in their career choice and to support them early at undergraduate medical school.

At the same time it is important to improve working conditions for future GPs in order to counteract the shortage of physicians especially in primary care. In an international study, the Commonwealth Fund interviewed GPs from 7 countries to compare aspects of their daily work and quality of health care: German GPs had a higher workload and were more dissatisfied with their work than their colleagues from other countries [23]. Germany is based on a Social Security Health care system and is funded by means of earmarked premiums. The system is more loosely organised than systems with National Health Services like in The United Kingdom, Spain and Sweden [24]. The German state has less influence and the system has a more pluralistic structure, with stronger influence of health care providers and social insurances. In systems with National Health Services, a referral from a GP is important for access to specialized care [25]. In contrast, GPs in Germany do not function as gatekeepers but patients have free access to ambulatory specialist services [25]. Moreover, there is no structured vocational training for GPs and vocational training has a poor image compared to other countries of Western Europe [26]. In Germany, physicians are able to choose training in any specialty at any time independent of their age or final grade. To take the necessary measures to ensure future primary health care in Germany it is highly relevant to identify the main factors influencing the career choice among doctors.

This study aims to explore the specialty medical students intend to choose and to identify factors influencing this choice. A special focus is laid on general practice. Therefore, the analysis focused on the identification of influencing factors differing among the students choosing a career in general practice and in other specialties.

## Methods

### Design of the study

The study was designed as a cross-sectional survey including all medical students in BW. All students studying at one of the 5 medical schools in BW (Freiburg, Heidelberg, Mannheim, Tuebingen and Ulm) were invited to participate in an online-survey between January 2010 and February 2010 independent of age, gender and academic year.

### Questionnaire

A questionnaire was developed based on a literature review [27]. Thereafter the technique of "concurrent think aloud" was used to ensure that the items were understandable and unambiguous. Four medical students, 2 vocational trainees, and 2 general practitioners were involved in this process. The technique of "concurrent think aloud" is a well-established qualitative method to monitor the meaning of questions by thinking aloud and addressing all comments and associations [28]. The initial questionnaire was piloted among 179 students. The final questionnaire comprised 27 items regarding possible key factors affecting students' career choice. Responses to the statements ("For me, it is important to...") were measured on a 5-point Likert scale ranging from 1 (fully agree) to 5 (fully disagree). Socio-demographic questions regarding age, gender and academic year were included. Moreover, the students were asked which medical specialty they aspire after finishing medical school. (free-text field). The final questionnaire could be requested from the authors.

### Data analysis

An exploratory factor analysis using the principal component method performed on the 27 items constituted 7 subscales which were determined by scree test and eigenvalues > 1. The solution was rotated using varimax rotation. The results of the factor analysis including the Kaiser-Meyer-Olkin measure of sampling adequacy, the factor loadings of the items and the internal consistency of the factors (Cronbach's alpha) can be found in Table 1. These 7 factors collectively explained 63% of the variance in the responses. After factor analysis we decided to divide the subscales in terms of content into occupational and individual aspects of career choice. The subscales 'Variety in job' (4 items), 'Patient-orientation' (2 items), 'Job-related ambition' (3 items) and 'Image' (4 items) cover the occupational aspects. The subscales 'Personal ambition' (2 items), 'Future perspective' (4 items) and 'Work-life-balance' (8 items) describe the individual aspects. For each subscale, a sum value was calculated from the corresponding items and linearly transformed to a scale ranging from 0 to 100 with higher scores indicating less importance. Furthermore a ranking of specializations favoured by students was created.

**Table 1:** Rotated factor loading^a ^with Kaiser-Meyer-Olkin measure for each of the 27 items of the questionnaire

Items	Factor I	Factor II	Factor III	Factor IV	Factor V	Factor VI	Factor VII	Kaiser-Meyer-Olkin
secure job			0.799					0.783

job with a future			0.710					0.830

flexible working hours	0.453							0.848

regular working hours	0.570							0.896

diversified working day					0.539			0.730

be less on night duty	0.657							0.872

good salary			0.498					0.794

secure income			0.729					0.787

join in research and development				0.790				0.718

deal with various diseases					0.545			0.735

up-to-date with research				0.732				0.766

preventive medicine						0.653		0.788

little physical stress	0.664							0.845

little mental stress	0.735							0.846

long-lasting relationships to patients						0.728		0.682

work in emergency medicine					0.649			0.761

broad medical knowledge					0.650			0.730

a lot of free time	0.641							0.845

separate professional and private life	0.477							0.842

part-time job	0.424							0.824

combine family and job							0.525	0.805

publicly appreciated		0.715						0.872

career goals^b^				0.482			0.459	0.750

private goals							0.771	0.708

positive reputation within medicine		0.848						0.788

positive reputation in the media		0.858						0.779

priority in medical training		0.639						0.893

Cronbach's alpha	.76	.81	.72	.59	.58	.56	.49	

Factor I 'Work-life balance'; factor II 'Image'; factor III 'Future perspective'; factor IV 'Job-related ambition'; factor V 'Variety in job'; factor VI 'Patient orientation'; factor VII 'Personal ambition'

^a ^Using an exploratory factor analysis; Extraction method: Principal axis factoring; Rotation method: Varimax with Kaiser normalization; Rotation converged in three iterations. Only loadings greater than 0.4 are shown.

^b ^contributes to factor III

Descriptive statistics including mean (M) and standard deviation (SD) of the transformed factor sums were calculated for specialty groups. Group comparisons between gender, year of study and specialty choice were analysed using chi-square test. Differences in the subscales between students choosing general practice and students choosing another specialty were examined using t-tests for independent groups. SPSS Version 18.0 (SPSS Inc., Chicago IL, USA) was applied for analysis. An alpha level of P < 0.05 was used for tests of statistical significance. However, as this was an exploratory analysis, *p *values are only descriptive in nature.

### Ethics approval

The ethics committee of the Heidelberg Medical School informed us that approval by an ethics committee was not necessary for a study which does not involve patient data. Anonymity of the participating students and data safety were ensured.

## Results

The online-questionnaire was answered by 1,299 students. Table 2 describes the characteristics of the sample. Nearly 60% of the study population were female. The mean age of the participants was 24.1 years (SD = 3.1). Considering a total population of 12,062 medical students [29] in BW, the number of participants corresponds to a proportion of 11%. No differences were found between the study population and the whole population of medical students in terms of gender (Table 2).

**Table 2:** Description of the sample

	Our sample **(n = 1,299)**^**1**^	Medical students in BW **(n = 12,062)**^**2**^
**Gender, n (%)**		
Female	774 (59.6)	7,180 (60.0)
Male	408 (31.4)	4,882 (40.0)

**Age, mean (SD)**	24.1 (3.1)	Data not available

**Year of study, n (%)**		Data not available
1^st ^- 2^nd^	304 (23.4)	
3^rd ^- 5^th^	688 (53.0)	
> 5^th^	173 (13.3)	

SD = Standard deviation, ^1^varies due to missing data, ^2 ^total sample of medical students in the federal state of BW (Baden-Wuerttemberg, Germany) for 2009/2010; Source: Statistical Office of the federal state of Baden-Wuerttemberg (Germany) [29]

While 406 students indicated a range of 20 different specialties as a future career choice, the majority of students (n = 708) indicated one of the following 6 specialties: 12% (152/1299) of the students wanted to become an internal specialist, followed by 10% (126/1299) intending to choose gynaecology and paediatrics, 9% (112/1299) favouring surgery and 8% (99/1299) anaesthesiology. General Practice was chosen by 7% (88/1299) of the students. Table 3, shows that students preferring general practice were on average 24.6 (SD = 3.3) years old which is comparable to the mean age of students choosing the 5 other specialties. 67% (59/88) of the students favouring general practice were female. A significant higher proportion of female than male students chose gynaecology and obstetrics (94%, 119/126) and paediatrics (81%, 101/125), whereas internal medicine (52%, 79/152) and anaesthesiology (49%, 48/99) were chosen by more male students (p < .01).

**Table 3:** Description of students with different choices of career^1 ^

	GP (n = 88)	Asth (n = 101)	Sur (n = 115)	Paed (n = 125)	Gyn (n = 127)	IM (n = 152)	other (n = 406)
**Gender, n (%)**							
Female	59 (67.0)	51 (50.5)	68 (59.1)	101 (80.8)	119 (93.7)	73 (48.0)	260 (64.0)
Male	29 (33.0)	48 (47.5)	44 (38.3)	24 (19.2)	7 (5.5)	79 (52.0)	146 (36.0)

**Age, mean (SD)**	24.6 (3.3)	24.9 (3.4)	23.2 (3.1)	23.9 (3.4)	24.1 (2.3)	24.2 (2.7)	24.1 (3.1)

**Year of study**							
1^st ^- 2^nd^	21 (23.9)	27 (26.7)	49 (42.6)	40 (32.0)	22 (17.3)	28 (18.4)	86 (21.2)
3^rd ^- 5^th^	48 (54.5)	63 (62.4)	54 (47.0)	57 (45.6)	84 (66.1)	91 (59.9)	257 (63.3)
> 5^th^	17 (19.3)	11 (10.9)	8 (7.0)	27 (21.6)	17 (13.4)	30 (19.7)	55 (13.5)

^1^varies due to missing data

SD = Standard deviation, GP = general practice, Asth = anaesthetics, Sur = surgery, Gyn = gynaecology and obstetrics, Paed = paediatrics, IM = internal medicine

All students, scored individual aspects lower (M = 12-15, SD = 13.1-14.1) and therefore rated them as more important than occupational aspects (M = 24-44, SD = 14.7-21.8), except for the subscale 'Work-life balance' (M = 40, SD = 15.6). The subscales 'Work-life balance', which included items regarding the extent of physical and psychological stress (see Table 1), and 'Image' (M = 44, SD = 21.8), which included items regarding public presentation of the specialty, were considered least important by all students. Table 4 demonstrates the main findings regarding individual and occupational aspects for student groups with different specialty choices.

**Table 4:** Importance of individual and occupational aspects in the future for various specialty choices (0 very important - 100 not at all important); M (SD)

	GP (n = 88)	Asth (n = 101)	Sur (n = 115)	Paed (n = 125)	Gyn (n = 127)	IM (n = 152)	other (n = 406)
**Individual aspects**							
*Personal ambition*	9 (11.9)	14 (16.0)	18 (16.7)	10 (12.9)	7 (9.3)	13 (13.1)	12 (14.4)
*Future perspective*	18 (13.9)	15 (11.9)	14 (12.4)	17 (12.9)	15 (12.5)	17 (14.5)	14 (12.5)
*Work-life balance*	37 (12.8)	44 (15.3)	46 (14.4)	37 (15.0)	36 (13.8)	43 (15.7)	40 (16.7)

**Occupational aspects**							
*Variety in job*	22 (12.9)	16 (13.2)	22 (14,9)	23 (12.4)	31 (13.2)	22 (13.9)	26 (15.5)
*Patient orientation*	19 (17.8)	51 (18.8)	47 (17.7)	32 (19.9)	33 (16.6)	39 (19.9)	42 (20.2)
*Job-related ambition*	39 (13.6)	33 (15.2)	28 (17.9)	34 (15.4)	37 (16.8)	31 (16.2)	29 (16.7)
*Image*	42 (20.8)	43 (21.3)	36 (22.5)	46 (19.2)	46 (21.2)	45 (21.6)	44 (22.3)

GP = general practice, Asth = anaesthetics, Sur = surgery, Paed = paediatrics, Gyn = gynaecology and obstetrics, IM = internal medicine; M = mean, SD = standard deviation

Students choosing general practice differed on all individual aspect subscales from students choosing other specialties (Table 5): For potential future GPs 'Personal ambition' and 'Work-life balance' were more important, while 'Future perspective' was rated less important. Within the occupational aspect subscales, 'Patient orientation' was considered the most important occupational aspect by students choosing general practice and was less important for students with other specialty choices (p < .05). 'Job-related ambition', on the other hand, was less important for students aiming for general practice (p < .05).

**Table 5:** Difference between students choosing general practice (GP) and students choosing another specialty in importance of individual and occupational aspects in the future (0 very important - 100 not at all important); M (SD)

	GP (n = 88)	all other (n = 1,026)	p*
**Individual aspects**			
*Personal ambition*	9 (11.9)	12 (14.2)	.02
*Future perspective*	18 (13.9)	15 (12.8)	.02
*Work-life balance*	37 (12.8)	41 (15.9)	.03

**Occupational aspects**			
*Variety in job*	22 (12.9)	24 (14.8)	.22
*Patient orientation*	19 (17.8)	41 (20.1)	<.01
*Job-related ambition*	39 (13.6)	31 (16.7)	<.01
*Image*	42 (20.8)	44 (21.8)	.46

*Statistical significances of difference: *p *< .05

## Discussion

This study evaluated preferences in career choice of students during medical school in Germany. Only 88 students (7%) want to become a general practitioner. This is a very low proportion considering the fact that currently about 14% of all physicians in Germany work in general practice and, thereof, about 25% are over 60 years [4]. However, previous studies have demonstrated, that the decision for a career as a GP often happens at a later point of time: either in an advanced academic year [13], in an advanced age [15,16] or in early residency [17].

Our data also show that specialty choices depend on gender. More female students in our study chose gynaecology or paediatrics and less female students chose internal medicine or anaesthesiology. This is in accordance with international studies showing that women are more interested in gynaecology or paediatrics than men [30-32]. Furthermore, it has been shown that being female and older age influences the choice for becoming a GP in a positive way [10-15,33]. Previous studies revealed that female physicians are influenced in their career choice by family-friendly working conditions and by the possibility, to have close relationships to patients, a sizeable income and a short residency. Male physicians, on the other side, are more often influenced by research and prestige [34].

Within our study we were able to demonstrate that students aspiring different medical specialties attach importance to different factors regarding career choice. In the total sample, 'Future perspective' and 'Personal ambition' were rated as most important reasons for specialty choice by students. The interaction between the choice of a career and having an interesting future with the specialty is an important factor which has impact on future choice [35]. Students mainly differed in the factor 'Patient orientation' being the most important for future GPs and least important for anaesthesiologists and surgeons. This result is concordant with previous studies [21,36].

Additional, compatibility of family and job is essential for further generations of physicians [7,17]. An own practice is especially attractive for female physicians who have already worked for a few years, as they have no night duties hospital anymore, working hours which are compatible with family, and a close physician-patient relationship [34]. General practice as well as gynaecology are specialties which offer the possibility of an own practice [4], students or young physicians might choose those specialties because of such opportunities.

In our sample, individual aspects such as 'Personal ambition' and 'Work-life balance' were more important for students aiming for general practice compared to students aiming for other specialties, whereas 'Future perspective' including the item "to have a good salary" was less important. Different international studies demonstrated that an assumed lower income is one reason why students hesitate to become a GP [5,6,18]. Concluding, students attaching high relevance to a high income later on are more likely to choose a specialty other than general practice.

Another difference between the two student groups was that the factor 'Patient orientation' was rated as more important by students choosing a career in general practice. This is in good agreement with previous studies showing that students who are interested in a close relationship to patients prefer general practice [10,20].

### Strengths and limitations

Strength of the presented study is that we used a validated questionnaire for the evaluation of career choice. Our established instrument of career choice for medical students is comparable to other existing international instruments such as the Specialty Choice Inventory Sci45 [37], for career guidance for specialty or the questionnaire from Wright et al. [38].

The respondents of our questionnaire are comparable regarding gender with the overall sample of medical students in BW [29]. A basic limitation of our online survey is that we can not calculate an exact response rate, because it is not sure whether all 12,062 students have received the invitation for the survey. Furthermore, due to the voluntary participation a selection bias in favour of students more interested in the issue of career choice (and probably more reflected) can not be excluded. As the study is based on a cross-sectional survey we cannot conclude causality in the analysis. Due to our research design, it was difficult to evaluate possible effects through experiences with general practice i.e. during practical training. There was no free text space for respondents to give reasons for their career choice. For further analysis a longitudinal design would be important to respond to these aspects and to identify reasons for choosing or not choosing a career in general practice. We had a low response rate regarding all medical students in the federal state (BW). Therefore, generalisation of our findings is limited. In addition, this was an exploratory study; *p *values should be interpreted carefully. Significant results might be due to chance and will need to be confirmed in further targeted studies.

## Conclusions

In general, the fact that only 1,299 medical students responded to the online survey should reinforce the need for medical schools to recognise that they should put more emphasis on career advice for undergraduates beginning at an early stage in medical training. The results of our study demonstrate that there are differences in the importance of factors between students aiming for a career in general practice and students interested in other specialties. Differences were particularly found in items concerning individual aspects and patient orientation. To attract more students to general practice those factors have to be addressed and to be made more explicit to medical students at an early stage in medical school. Furthermore, conditions for working as a GP have to be changed according to the expectations of the coming generations of physicians. It should be a joint effort by physicians as well as at a political level to present the core craft skills of GPs as complex and valuable to medical students and teaching those systematically to vocational trainees. As a final remark, a professional pride in doing a challenging job well should be more reflected and shown by physicians which could have an essential impact on future recruitment.

## Competing interests

The authors declare that they have no competing interests.

## Authors' contributions

KK and KG designed the study and collected data. KK conducted initial data analyses, drafted and revised the manuscript. KG, AM, KH and SJ contributed to interpretation of the data and revision of the manuscript. AL and JS critically revised the manuscript for important intellectual content. All authors read and approved the final manuscript.

## Pre-publication history

The pre-publication history for this paper can be accessed here:

http://www.biomedcentral.com/1471-2296/12/25/prepub
